# Framework for classifying chemicals for repeat dose toxicity using NAMs

**DOI:** 10.1007/s00204-025-04069-1

**Published:** 2025-05-24

**Authors:** J. E. Doe, P. Botham, D. Holland, M. Fuart Gatnik, V. Giri, H. Kang, P. Kalra, S. León Pérez, S. Marty, S. Moors, R. Raeburn, E. Reale, R. Settivari, M. Sica, K. Z. Travis, S. J. Wijeyesakere

**Affiliations:** 1 School of Pharmacy and Biomolecular Sciences, Liverpool John Moores University, Byrom Street, Liverpool, L3 3AF UK; 2 Syngenta, Jealott’s Hill International Research Centre, Bracknell, Berkshire RG42 6EY UK; 3 ExxonMobil Petroleum and Chemical, 1831 Machelen, Belgium; 4 Merck d.o.o., Letališka Cesta 29C, 1000 Ljubljana, Slovenia; 5 BASF SE, Experimental Toxicology and Ecology, Carl-Bosch-Str 38, 67056 Ludwigshafen am Rhein, Germany; 6 LyondellBasell Industries Holdings B.V., 3013 AA Rotterdam, Netherlands; 7 Simulations Plus Inc., Lancaster, USA; 8 ECETOC AISBL, Rue Belliard 40, 1040 Brussels, Belgium; 9 Dow Chemical Company, Toxicology and Environmental Research and Consulting, 1803 Building, Midland, MI 48667 USA; 10 BASF Personal Care and Nutrition GmbH, Henkelstrasse 67, Düsseldorf, Germany; 11 Afton Chemical Limited London Road, Bracknell, Berkshire RG12 2UW UK; 12 Nestlé Research, Société des Produits Nestlé SA, Vers-Chez-Les-Blanc, 1000 Lausanne 26, Switzerland; 13 Corteva Agriscience, Haskell R&D Center, Newark, DE 19711 USA; 14 Evonik Operations GmbH, 45127 Essen, Germany; 15 RSA, Largs Yacht Haven, Irvine Road, Largs, KA30 8EZ UK

**Keywords:** Chemical safety assessment, Classification and labelling, New approach methodology (NAM), Regulatory toxicology, Risk management, Next-generation safety assessment

## Abstract

EPAA’s ‘NAM Designathon 2023’ challenge for human toxicity sought to identify a classification system capable of categorising chemicals based on their bioactivity and bioavailability properties determined using non-animal methodologies (Worth et al. 2025). The proposal is made to classify chemicals into three levels of concern: low concern could be used without restriction, medium concern requiring assessment to establish safe use levels and high concern being candidates requiring risk management (Berggren and Worth in Regul Toxicol Pharmacol 142:105431, 10.1016/j.yrtph.2023.105431, 2023). We developed a NAMs based classification system for “human systemic toxicity” mainly focussed on repeat dose toxicity, similar to the assessment carried out in classification for ‘Specific Target Organ Toxicity—Repeated Exposure’ (STOT-RE) based on ECETOC’s Tiered Approach integrating three lines of evidence: In silico predictions, In vitro bioavailability and PBK modelling, In vitro bioactivity assays. The first stage employed an in silico approach, covering several toxicity endpoints across various (Q)SAR in silico models to identify indicators of toxicity. Bioavailability was categorised by simulating 14-day plasma *C*_max_ predictions for a standard dose level using three TK models (Firman et al. in Arch Toxicol 96:817–830, 10.1007/s00204-021-03205-x, 2022). Bioactivity was categorised using a matrix with potency and severity. In vitro data were obtained from ToxCast. Potency makes use of dose response AC50 values. Severity categorisation is based on consideration of the adverse effects associated with the assays. 12 chemicals have been assessed through the framework. Overall, we have demonstrated that the matrix suggested by the EPAA Designathon can be used to categorise chemicals into three different levels of concern but there are areas still to be explored especially for the range of assays used, the framework categorisation being defined, and how such a matrix would fit into a tiered approach, pragmatically, including targeted in vivo studies.

## Introduction

The European Centre for Ecotoxicology and Toxicology of Chemicals (ECETOC) developed a framework for the use of new approach methodologies (NAMs) in assessing the safety of chemicals in Ball et al. (2022). The ECETOC framework is a tiered approach using the Threshold of Toxicological Concern TTC (Tier 0), in silico assessment (Tier 1), in vitro assessment (Tier 2) and targeted in vivo studies (Tier 3) which includes hazard characterisation, exposure assessment and risk assessment.

The framework became one of the drivers behind the idea of the European Partnership for Alternative Approaches to Animal Testing (EPAA) to promote a Designathon which called for ideas and concepts on how NAMs could be used for the classification of chemicals for “human systemic toxicity” (EPAA 2023; Worth et al. 2025). The goal was to develop a classification scheme that incorporated NAMs whilst giving similar levels of protection as the current schemes based on conventional animal studies but not necessarily to reproduce the existing schemes. This scheme includes a range of outcomes related to “systemic toxicity”; however, the Designathon intentionally left its interpretation ambiguous.

The pilot phase of the Designathon launched on May 31, 2023, the challenge being to develop an approach using NAMs (In this case NAM implying non-animal methods) capable of assigning chemicals into three levels of concern:

- Low (L)—presumed to be non-hazardous—no further data required, can be used widely.
- Medium (M)—hazardous chemicals—Health-based guidance values (HBGVs) required, more information is needed to verify safe use.
- High (H)—chemicals of high concern—restrict use unless additional data can be provided to change the category

**Fig. 1:**
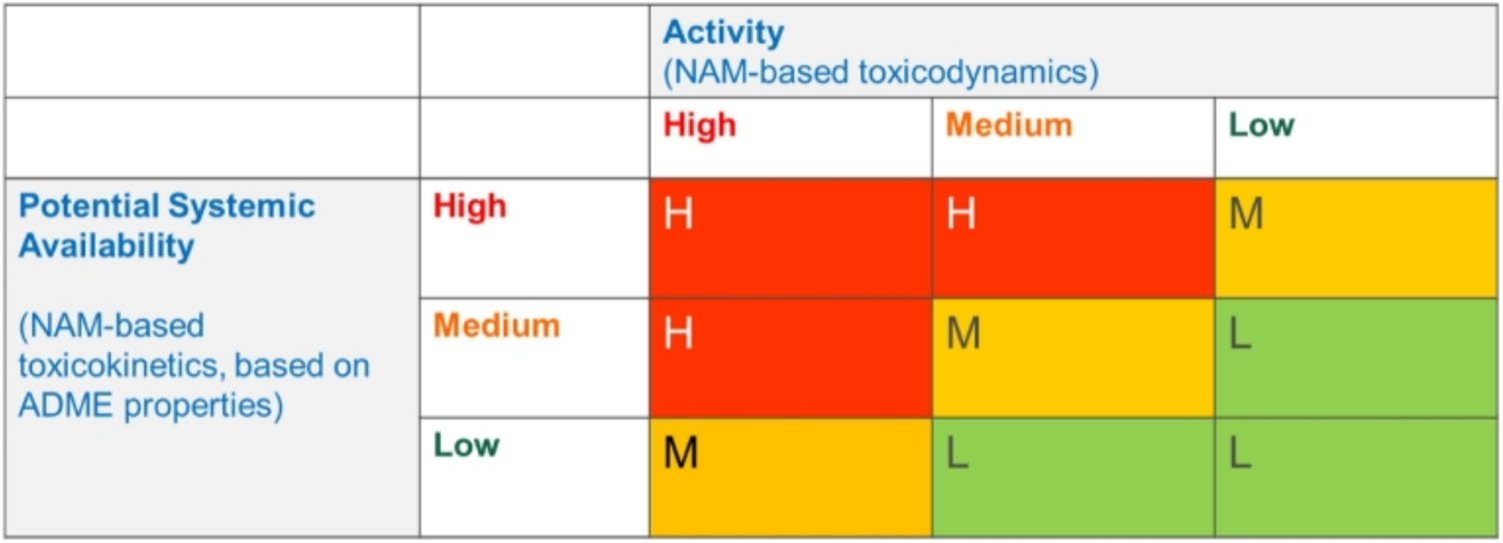
Kinetics and dynamics matrix used in EPAA’s Designathon. Reprinted with permission from “Berggren E, Worth AP. Towards a future regulatory framework for chemicals in the European Union—Chemicals 2.0. Regul Toxicol Pharmacol. 2023;142:105431. 10.1016/j.yrtph.2023.105431”. Copyright © 2023 The Authors, Elsevier. Original figure description: A new classification scheme for chemicals based on three levels of concern (High, Medium and Low). (Berggren and Worth 2023)

The EPAA Designathon suggested a matrix that assesses the ability of a chemical to cause adverse biological changes, toxicodynamics (TD), and the ability to reach the target site, toxicokinetics (TK), to decide the overall categorisation as shown in Fig. 1.

A list of 150 chemicals has been provided by the EPAA. The list was chosen by scientists at the Joint Research Centre (JRC) to have an equal number of chemicals in each category (L, M and H).

However, the category for each chemical was not revealed nor were any data on the chemicals supplied. The aim of this exercise was not to correctly classify the chemicals, but to present a hypothesis on how to classify all, or a fraction of, these chemicals into the three aforementioned categories.

ECETOC decided to enter the Designathon using the framework presented in Ball et al. (2022) as a basis for its contribution. The EPAA Designathon aimed to encourage methods that do not use laboratory animals, thus aligning with Tiers 0–2 of the framework.

In ECETOC’s contribution to the Designathon, we interpreted “human systemic toxicity” to be mainly focussed on repeat dose toxicity, similar to the assessment carried out in classification for Specific Target Organ Toxicity—Repeated Exposure (STOT-RE) (ECHA 2023a, b). We did not attempt to assess mutagenicity, carcinogenicity or reproductive toxicity in the classification although indicators of these toxicities were included for the in silico assessments. The endpoints designated as STOT-RE, mutagenicity, carcinogenicity or reproductive toxicity are manifestations of one or more modes of action of a chemical and it would be expected that there would be some interrelationship between the endpoints. We have explored this relationship in “Discussion” of this paper.

We aimed to include a wide range of chemicals with differing levels of concern and a diverse array of in vitro data. We first searched for chemicals that have an adequate data set in ToxCast (USEPA 2024), based on various factors, like the amount of endpoints tested. We then evaluated the conventional repeat dose toxicity data on the chemicals to give an indication of their level of concern (LoC) and selected 4 which we considered to be of high concern, 4 of mid-concern, and 4 of low concern. On this basis, we selected and evaluated 12 chemicals from the EPAA Designathon list: nitrobenzene (98-95-3), ouabain (630-60-4), benzoic acid (65-85-0), safrole (94-59-7), 2,4,6-tri-tert-butylphenol (732-26-3), phenol (108-95-2), 1-chloro-4-nitrobenzene (100-00-5), colchicine (64-86-8), 4-nitrophenol (100-02-7), diethylphthalate (84-66-2), carbaryl (63-25-2) and chlorpropham (101-21-3). The category to which the chemicals had been assigned was not used in the evaluation, but the categories derived from the framework and from conventional studies were compared after the assessments were concluded.

Our evaluation was based on tiers 1–2 of the ECETOC framework for hazard assessment and included an assessment of bioavailability to follow the Designathon classification scheme. Risk assessment was not part of the EPAA’s original brief (EPAA 2023), which was focussed on hazard characterisation, although it is a key element of the ECETOC framework where exposure assessment and hazard assessment are carried out in an integrated way (Ball et al. 2022).

## Overview of the process

The EPAA’s ‘NAM Designathon 2023’ challenge for human toxicity seeks to identify classification systems capable of categorising chemicals based on the intrinsic toxicodynamic and toxicokinetic properties. This initial proposal of a NAMs based classification system was based on a matrix incorporating bioactivity (TD) and systemic bioavailability (TK).

**Fig. 2:**
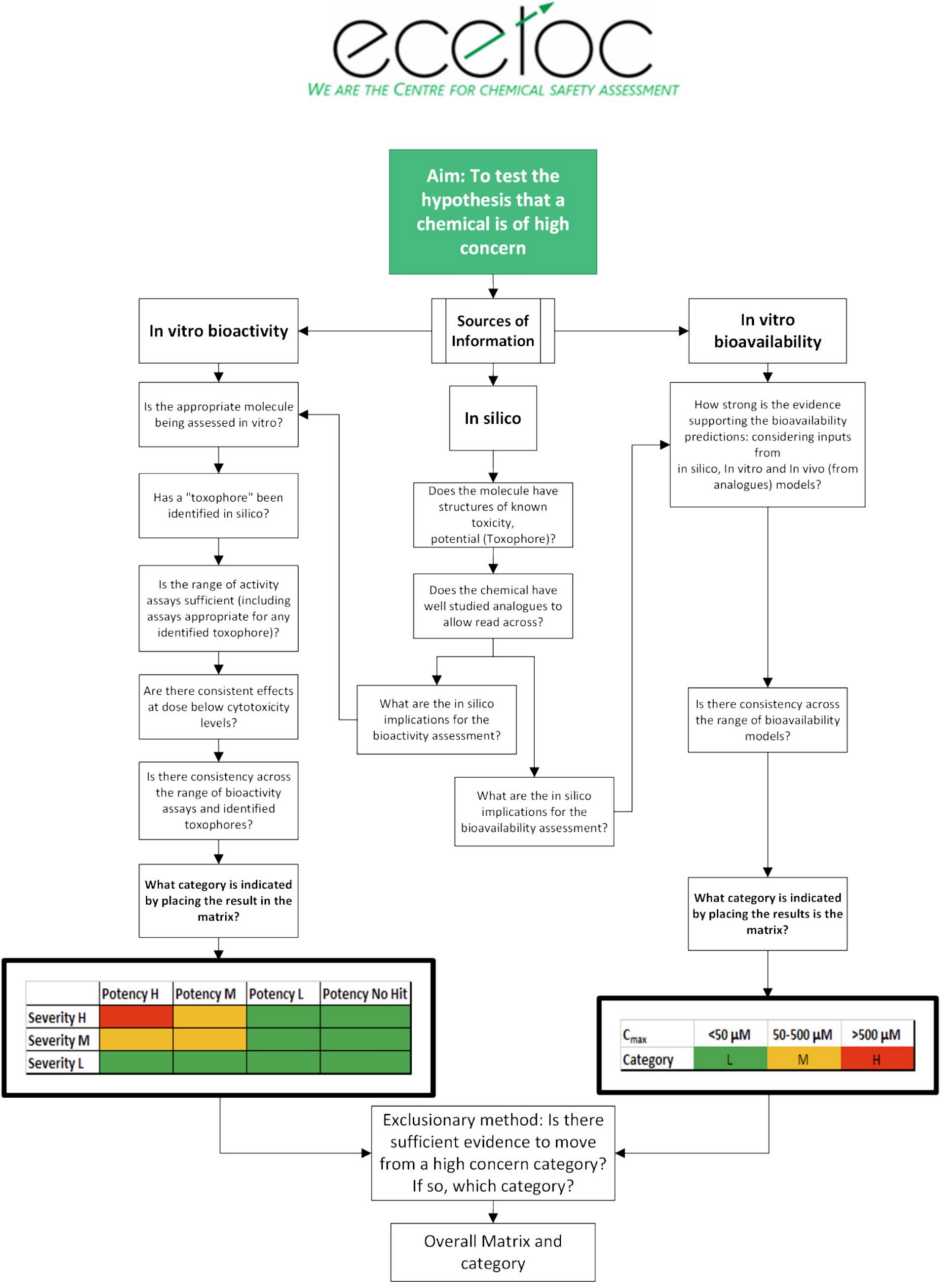
Outline of the assessment process

Accordingly, to populate the final EPAA TD/TK matrix, we have developed a process, outlined in Fig. 2 that employs.

- In silico assessments, including likely metabolite identification
- In vitro bioavailability and physiologically based PBK modelling
- In vitro bioactivity assays

First, we have used a range of in silico tools to identify indicators of potential toxicity and likely metabolites. This information is used to provide a preliminary assessment and to review the adequacy of the in vitro database (“In silico assessment”).

We used in vitro bioactivity data to generate a matrix that incorporates potency based on dose–response (AC50 values) and severity of the assay for the indicated effect (“Bioactivity assessment’).

When it comes to bioavailability, we have developed a process based on 14-day plasma level calculation. The level of concern for bioavailability for the EPAA matrix is determined using a standard dose level leveraging in vitro and in silico TK data to predict the maximum plasma concentration *C*_max_ (“Bioavailability assessment”).

The general approach of the framework is based on the hypothesis that all chemicals are initially of High concern. Afterwards, information is evaluated to determine if there is sufficient evidence to move the chemical to Medium or Low concern. The framework presented should not be considered a decision tree but rather as a structured assessment of evidence. This assessment takes into account both the quality of the evidence and the information provided by it. The conservative nature of the framework ensures that a chemical remains as high concern if there is insufficient evidence to move it to medium or low concern, or if sufficient evidence confirms its status as high concern.

## In silico assessment

**Fig. 3:**
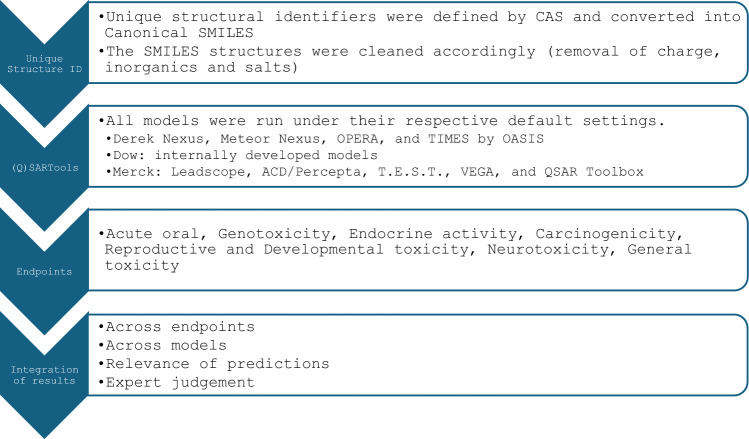
General workflow for the in silico assessment used in the framework

Tools and models applied are shown in Appendix A, a short summary of the general workflow is shown in Fig. 3 (Derek Nexus 2023, Meteor Nexus 2023, OASIS TIMES 2023, Tox Suite 2023, Impurity Profiling Suite 2023, Leadscope 2023).

The use of Quantitative structure–activity relationship models ((Q)SAR) tools for predicting toxicological properties within the context of NAMs has become a standard approach. It is recognised that consistent predictions of the same endpoint across different (Q)SAR models, ideally, utilising diverse training sets and modelling methodologies, can enhance confidence in predictions. This confidence is further strengthened when each individual model’s predictions are deemed reliable and relevant.

In addition, in the selection of models to qualify for application within the proposed approach, it is recommended that (Q)SAR models be rigorously selected based on their scientific and, ideally, regulatory validity, as well as their availability and ease of use, particularly concerning automation and integration.

For this analysis, several (Q)SAR tools were utilised, including both expert rule-based and statistical based (Q)SAR prediction methodologies. Rule-based (Q)SAR models are built on expert knowledge that is used to group chemicals according to a property, this results in a qualitative prediction based on the presence or absence of a structural feature. A statistical (Q)SAR model is built using model-dependent regression and classification methods, and this results in an often-quantitative correlation between a chemical structure and toxicity. Results from these models provide binary classifications, which usually do not address potency. Detailed information about the tools used is available in Appendix A.

Since current (Q)SARs do not support predictions for “systemic toxicity”, endpoint selection for toxicological evaluations was guided by conservative assumptions. Therefore, we selected available and validated (Q)SAR models that could be indicative of systemic toxicity. This selection included models for carcinogenicity, mutagenicity, reproductive and developmental toxicity, endocrine activity, neurotoxicity, general toxicity, and some organ-specific toxicity. In addition, models predicting acute oral toxicity were also included. The aim was to capture a wide range of indications of toxicity that could be subsequently followed up by in vitro bioactivity assays.

The (Q)SAR models were applied in a semiautomated manner, meaning that the selected chemicals were processed through these models with considerations for reliable predictions, whilst facilitating their application in an automated, screening setting. The tools were run under their respective default settings. For predictions to be used as indicators of toxicity the following was taken into account: consistency across multiple models, any applicability domain information when available, as well as the reliability and relevance of predictions to repeat dose toxicity. Here, relevance refers to both if a model is suitable to assess the endpoint being investigated, i.e. repeat dose toxicity, as well as its relevance of application within this framework.

**Fig. 4:**
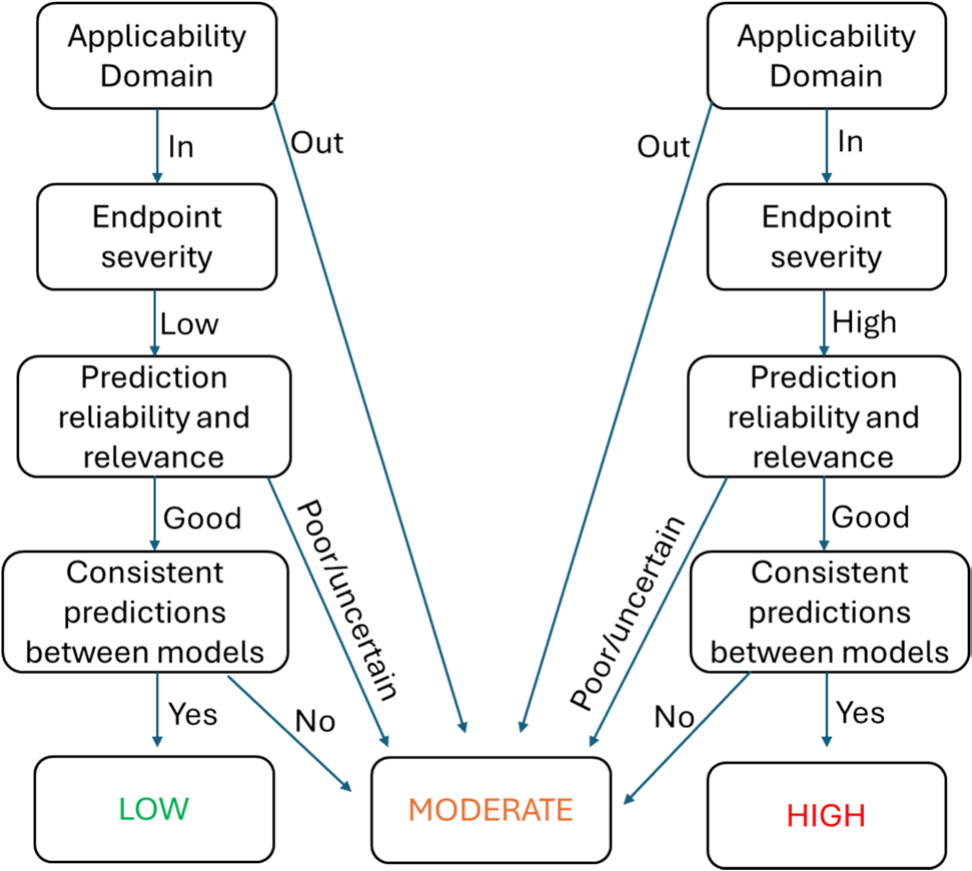
A decision framework for in silico output level of concern assignment. This approach was applied to each model used before reaching a final assignment of severity for a given chemical. Note: This is a rough guide, and expert knowledge sometimes overrode the framework

Toxicity indications for each substance were assessed using a predefined scheme that categorised predictions into three levels based on expert review: strong, moderate, and low indication of toxicity as shown in Table 1. As demonstrated in Fig. 4, a strong indication of toxicity was assigned to chemicals that showed positive predictions across several evaluated endpoints, demonstrated consistency across different models, fell within the applicability domain, and were deemed reliable and relevant by experts. Conversely, a no or low indication of toxicity was attributed to chemicals where predictions were predominantly negative or a lack of positive, models consistently indicated no toxicity, and the predictions were assessed as reliable and relevant by the experts. In cases where an overall indeterminate conclusion was reached by an expert regarding all the evaluated criteria, a moderate indication was noted. These indeterminate outcomes pertain to situations where not all models fell outside their applicability domains, not all predictions demonstrated poor reliability and relevance, and the consistency amongst models did not encompass all available models. Since in silico methods were considered in the initial evaluation step and as one line of evidence, a conservative approach was adopted to ensure that potentially valuable information from the in silico assessments was not overlooked.

**Table 1:**
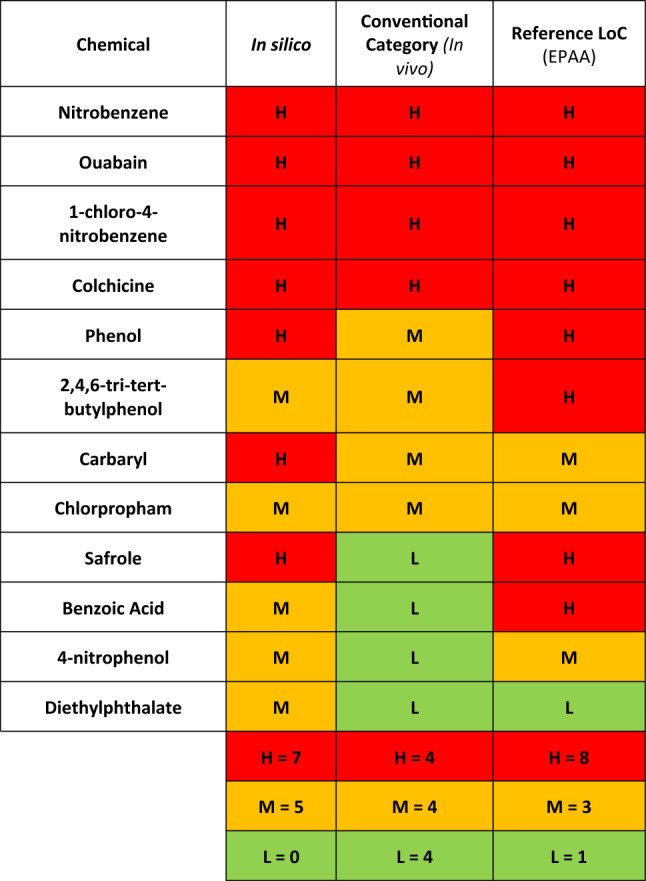
Comparative analysis for the 12 chemicals.

From left to right, in silico assessment, ‘conventional category’ literature level of concern using in vivo data and ‘Reference LoC’ EPAA literature level of concern based on “systemic toxicity”

The majority of substances were attributed to the high and moderate indication of toxicity levels. There was no substance assigned to the low indication level from in silico prediction. This is in part because *some* in silico models may be conservative by nature and a lack of alert does not mean a negative prediction, but it could also be a knowledge gap in the model. As a result, two intermediate categories were introduced for chemicals falling into the moderate category: a moderate/high was assigned to safrole, and low/moderate was assigned to benzoic acid. These intermediate categories were used when the expert assignment to one of the three main categories was challenged by uncertainties due to limited consistency amongst the results and underpinned model and prediction reliability and relevance. For these two instances, the conservative option was selected to move forward, this being high for safrole and moderate for benzoic acid.

In concluding the analysis, a conservative approach was adopted, and worst-case assumptions were applied where applicable.

Comparing the results from the analysis above with in vivo studies revealed that in silico methodology adopted in this exercise generally yielded more conservative evaluations (Table 1). Nonetheless, there was an alignment for all substances identified as high concern based on in vivo results. For substances categorised as moderate from in vivo studies, the in silico methodology yielded either moderate or high indications. For most substances rated as low concern in in vivo, a moderate indication was assigned, except for safrole which was high. It is also noteworthy to mention that many of the chemicals used in this work could also be present in the training sets of some models, and this is due to them being well-known chemicals with rich toxicological data. If the chemical is well represented in the training set, the toxicity indication from the in silico methodology should be given further consideration as the amount and quality of the data used in the assessment is expected to be applicable. These observations could impact the general reliability of in silico predictions when applied to a novel chemical.

In fact, it is possible that no reliable and relevant conclusion can be drawn, or irrelevant positive or negative outcomes are obtained based on (Q)SAR predictions. The approach proposed by the authors emphasises an integrated strategy that combines in silico and in vitro methods to generate diverse lines of evidence, recognising that (Q)SAR predictions will not be used as single line of evidence. It additionally suggests that when the generated information supports an informed decision, conclusions can be drawn; however, if no such conclusion can be reached based on the available evidence, more targeted testing strategy can be designated.

## Bioactivity assessment

Bioactivity was categorised using a matrix incorporating both potency and severity. The matrix for in vitro bioactivity data incorporates dose response (AC50) to assess potency and the severity of the effect indicated by the assay. For instance, assays related to oestrogenic receptor activity could lead to a range of adverse effects and would be rated as High, whilst assays related to peroxisome proliferator-activated receptor binding (PPAR) would be rated as Low as they lead to a specific effect of disputed relevance (Peraza et al. 2005). The severity of the effect was determined from the descriptors of the assay within the ToxCast reports. Examples of the rating of assays are shown in Table 2. It should be noted that this is a somewhat subjective process, bit it was included in an attempt to bring severity into the assessment.

**Table 2:** Results for the active assays of the major metabolite chloroaniline of the parent molecules chloronitrobenzene

Assay name	Target family	Target subfamily	Biological process target	Detection technology type	Tissue	Implied severity	Potency in assay
CEETOX_H295R_MTT_cell_viability	Cell cycle	Proliferation	Cell death	Fluorescence	Adrenal gland	H	M
CEETOX_H295R_DOC	Steroid hormone	Glucocorticoids	Regulation of steroid hormone biosynthetic process	Spectrophotometry	Adrenal gland	M	L
CEETOX_H295R_OHPROG	Steroid hormone	Progestagens	Regulation of steroid hormone biosynthetic process	Spectrophotometry	Adrenal gland	M	L
CEETOX_H295R_PROG	Steroid hormone	Progestagens	Regulation of steroid hormone biosynthetic process	Spectrophotometry	Adrenal gland	M	L
OT_FXR_FXRSRC1_1440	Nuclear receptor	Non-steroidal	Protein stabilisation	Fluorescence	Kidney	M	M
OT_NURR1_NURR1RXRa_1440	Nuclear receptor	Non-steroidal	Protein stabilisation	Fluorescence	Kidney	M	M
TOX21_ERR_Antagonist	Nuclear receptor	Orphan	Regulation of transcription factor activity	Luminescence	kidney	L	L
TOX21_ERa_BLA_Antagonist_ratio	Nuclear receptor	Steroidal	Regulation of transcription factor activity	Fluorescence	Kidney	L	L
TOX21_ERb_BLA_Antagonist_ratio	Nuclear receptor	Steroidal	Regulation of transcription factor activity	Fluorescence	Kidney	L	L
T + D4:D20OX21_AhR_LUC_Agonist	DNA binding	Basic helix–loop–helix protein	Regulation of transcription factor activity	Luminescence	Liver	M	L
LTEA_HepaRG_UGT1A1	Transferase	Glucuronosyltransferase	Regulation of transcription factor activity	Fluorescence	Liver	L	L
LTEA_HepaRG_CYP1A2	Cyp	Xenobiotic metabolism	Regulation of transcription factor activity	Fluorescence	Liver	L	L
LTEA_HepaRG_CYP1A1	Cyp	Xenobiotic metabolism	Regulation of transcription factor activity	Fluorescence	Liver	L	M
ACEA_AR_agonist_AUC_viability	Cell cycle	Cytotoxicity	Cell proliferation	Label-free technology	Prostate	H	M
ACEA_AR_antagonist_AUC_viability	Cell cycle	Cytotoxicity	Cell proliferation	Label-free technology	Prostate	H	M
ACEA_AR_antagonist_80hr	Nuclear receptor	Steroidal	Cell proliferation	Label-free technology	Prostate	H	M
BSK_hDFCGF_Proliferation	Cell cycle	Proliferation	Cell proliferation	Spectrophotometry	Skin	H	M
CCTE_Simmons_AUR_TPO	Oxidoreductase	Peroxidase	Regulation of catalytic activity	Fluorescence	Thyroid gland	L	M
BSK_3C_Proliferation	Cell cycle	Proliferation	Cell proliferation	Spectrophotometry	Vascular	H	L

The first six columns are the ToxCast descriptors for each active assay and are used to determine the ‘implied severity’ in the sixth column as H(igh), M(edium) or L(ow). ‘Potency in assays’ was derived from bioactivity assessments of AC50 values as H(igh), M(edium) or L(ow)

The AC50 values determined by the in vitro assays are placed into one of three categories in the matrix, High, Mid and Low. The boundaries of the categories were based on experience of using in vitro assays which indicated that a range of two orders of magnitude would be required for the Mid-category because of the wide range of values. The lower limit of High at 0.1 µM and the upper limit of Low 10 µM were set somewhat arbitrarily. The intention was to first assess a number of chemicals using these limits and then to review the categories that resulted and amend the limits as necessary, as described in “Sensitivity analysis”:

High < 0.1 µM; Medium 0.1 µM to 10 µM; Low > 10 µM.

The in vitro dose–response curves are reviewed to ensure confidence in AC50 values. Next, the results of each assay are placed into a bioactivity matrix (Table 3) with severity in one axis and AC50 categories on the other including no activity hits. The matrix output is then used to determine the level of concern of the chemical to be taken forward into the overall matrix.

**Table 3:**
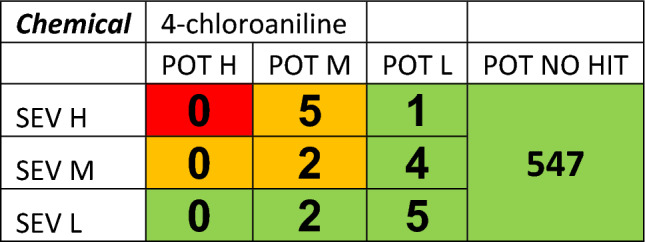
Bioactivity matrix for metabolite 4-chloroaniline, showing the number of active assays with specific degrees of potency and severity (potency = ‘POT’ and severity = ‘SEV’)

In order to explain the bioactivity process, we will use chloronitrobenzene as an example. The initial in silico assessment placed it in the High level of concern category and highlighted a major metabolite chloroaniline as well as a close mechanistic analogue aniline. Both findings were also confirmed by a follow-up literature research. Based on this information, it was decided that the bioactivity of both the parent compound and its metabolite needed to be assessed.

Chloronitrobenzene showed low to no activity in most assays, whilst the major metabolite chloroaniline showed activity in a wider range of assays (Table 2); the resulting activity matrix is shown in Table 3.

Once the assay data have been placed in the matrix and initially assessed, the worst-case potency is selected as a conservative starting assumption. An evaluation is then made of the results using a set of questions as shown in Table 4. The way these results are used with lines of evidence from the in silico and bioavailability assessments are discussed in further detail in “Overall assessment”.

**Table 4:**
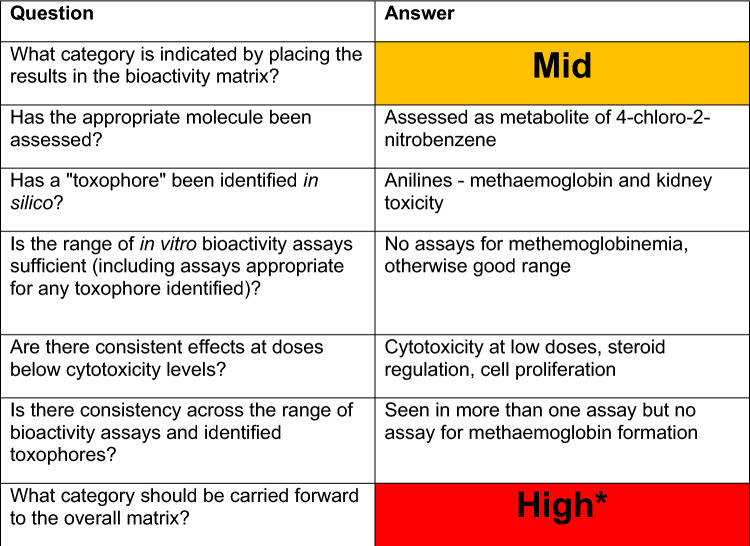
Evaluation of the results for metabolite chloroaniline (parent chemical chloronitrobenzene)

*Considered to be High as insufficient evidence because of lack of appropriate assays

The same bioactivity evaluation is performed for all remaining chemicals, and the full results can be seen in Table 8.

## Bioavailability assessment

Bioavailability indicates the extent to which a chemical enters the systemic circulation and becomes available for biological processes in a given species. It is, therefore, a crucial component of the NAM-based classification system developed for the EPAA’s Designathon. The bioavailability of chemicals varies depending on the route of administration, physico-chemical and toxicokinetic properties, as well as the physiology of the organism.

ECETOC’s contribution interpreted “potential systemic bioavailability” as the maximum plasma concentration (*C*_max_ in µM) predicted by PBK modelling after a 14-day repeat dose scenario for a chemical with an oral exposure. For this work, we limited the simulation to the oral route, but it would be possible to develop similar simulations for other routes such as inhalation and dermal if the physico-chemical properties and use profile indicated that would be necessary.

The simulated exposure scenario was a 1 × daily oral dose of 0.1 mmol/kg body weight, dosed as a solution for 14 days. In all the cases, the physiology PBK model was of a 70 kg healthy American male. This approach was chosen to provide a convenient way to derive one value for bioavailability that could be used in the matrix. An estimate of *C*_max_ after 14 days of dosing was selected as it allowed for the effects of possible prolonged half-life and/or accumulation to be reflected in one value. The choice of dose units makes a clean break from the traditional mg/kg approach whilst aligning with in vitro norms—it also does not penalise chemicals for having a high molecular weight. Dose levels were selected to be reasonably conservative, with a much higher than typical human chemical exposures and a higher than typical pharmaceutical dose. However, the dose is still lower than the 1000 mg/kg limit for repeat dose in animal studies. Dose equivalents are presented in Table 5.

**Table 5:** Dose equivalence of mmol/kg and a range of molecular weights

		Molecular weight 50 g/mol		Molecular weight 250 g/mol		Molecular weight 600 g/mol	
mmol/kg	mmol	mg/kg	g/person	mg/kg	g/person	mg/kg	g/person
0.1	7	5	0.35	25	1.75	60	4.2

Three PBK modelling tools were selected—httk, PK-Sim®, and GastroPlus®—to ensure a broader representation of modelling strategies and default assumptions across platforms commonly used in regulatory and research settings. The httk model, developed by the US EPA, is open-source, optimised for high-throughput simulations, and well suited for batch processing of large chemical inventories. PK-Sim® and GastroPlus®, on the other hand, are more mechanistic, with detailed physiological models, particularly for oral absorption processes. This variety allowed us to compare predictions across models that differ in complexity, assumptions, and treatment of parameters like absorption, metabolism, and clearance. By including models with differing levels of granularity and accessibility, we aimed to capture a realistic range of predicted *C*_max_ values and assess how model selection influences categorisation.

These models used a standardised human physiology (70 kg, American adult male) and their default settings for some of the toxicokinetic processes, e.g. intestinal absorption and renal clearance. The models were run with a minimal set of chemical-specific input data. Wherever available, the measured in vitro intrinsic hepatic clearance (CL_int_) and fraction unbound plasma (*f*_up_) data reported in the ToxCast database were used as input parameters for the PBK models. These parameters were scaled appropriately using in vitro to in vivo extrapolation (IVIVE) approaches. In the absence of the experimental values to predict the bioavailability of the compounds, in silico predicted CL_int_ and *f*_up_ values were used.

**Fig. 5:**
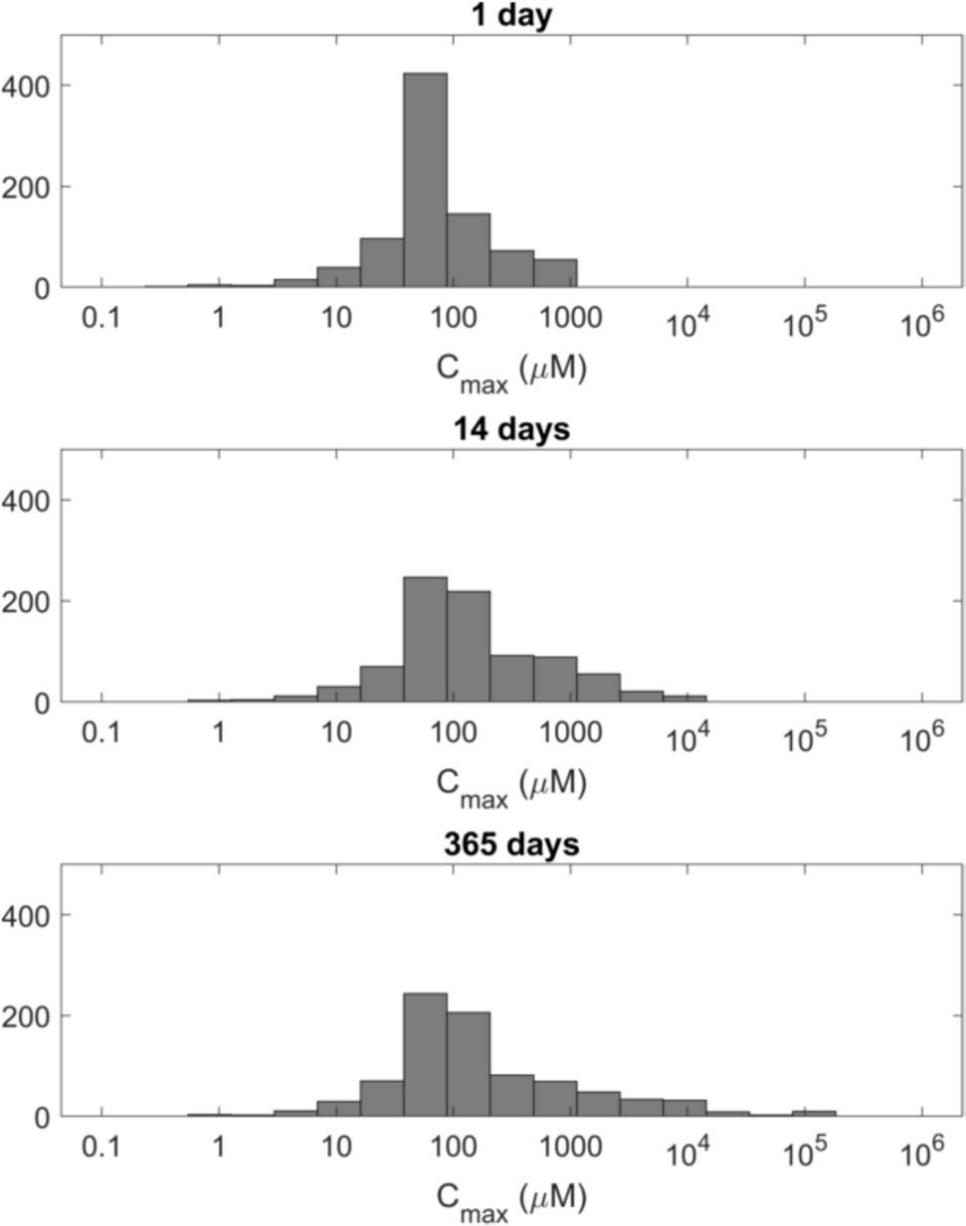
Comparisons of the frequency distributions of *C*_max_ values on a log scale for 1-, 14-, and 365-day simulation periods

The httk model suite from the US-EPA is designed to handle a large number of chemicals simultaneously. We ran the PBK model for all 857 chemicals with measured in vitro chemical-specific inputs available in the underlying httk database. The impact of selecting a 14-day simulation period is demonstrated by comparing the frequency distributions for 1-, 14-, and 365-day simulations, as shown in Fig. 5. As expected, the upper tail of the exposure distribution extends with longer exposure periods (28 and 365 days); however, the difference between the distributions for 14 and 365 days of exposure is not significant. The observed minimal difference between these distributions supports the choice of a 14-day simulation period as the endpoint for our analysis.

Chemical categorisation for bioavailability was based on the overall results for the 14-day simulation run in httk for the 857 chemicals. We selected *C*_max_ values of 50 and 500 µM as cutoff values, which divide the 857 chemicals from the httk database into approximately 25% below 50 µM (Low), about 50% between 50 and 500 µM (Mid), and around 25% above 500 µM (High).

From the analysed chemicals, only benzoic acid had a PBK model published (Hoffman and Hanneman 2017). However, as the approach in this work required fit for purpose models uniform across a variety of chemicals, this model structure was not chosen for our evaluation. Nevertheless, in vitro CL_int_ and *f*_up_ were available for 8 of the 12 chemicals selected for our evaluation (nitrobenzene, safrole, phenol, colchicine, 4-nitrophenol, diethylphthalate, carbaryl and chlorpropham), whilst they were unavailable for the other four (ouabain, benzoic acid, 2,4,6-tri-tert-butylphenol and chloronitrobenzene), preventing a completely uniform approach for all chemicals.

Sipes et al. (2017) used in silico methods to estimate the required model inputs for a large number of chemicals. These same inputs were used to run the three models for the four chemicals lacking in vitro input data. The resulting *C*_max_ estimates were multiplied by a commonly used (Dankovic et al. 2015) uncertainty factor of three to allow for the additional uncertainty introduced using in silico phys-chem and clearance model inputs rather than in vitro inputs. This factor was selected based on comparative accuracy of kinetic models based on in vitro and in silico data, when evaluated against in vivo data. Importantly, this adjustment is not intended to imply that predictions based on in vitro data are free from uncertainty; rather, it is an effort to maintain a comparable level of uncertainty across all twelve chemicals. Whilst a literature review could potentially refine this factor, such analysis was beyond the scope of the current study.

In cases where using the three different PBK models resulted in substances being placed into different categories, the worst-case category was selected as a suitable conservative assumption of the overall bioavailability classification. The results for all 12 substances are presented in Table 6.

**Table 6:**
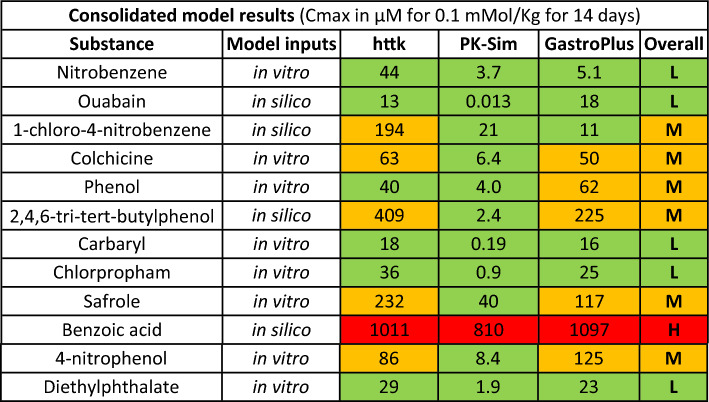
Classification of bioavailability for the 12 selected chemicals

The overall classification is the worst-case amongst the three categories determined using three different PBK models (httk, PK-Sim, GastroPlus). High > 500 µM (red); Mid 500–50 µM (orange); Low < 50 µM (green)

The three PBK models were run using their respective default approaches wherever possible. Due to inherent differences between model structures, variations in *C*_max_ predictions are inevitable. For instance, httk defaults to 100% oral bioavailability (although a measured or estimated value can be provided as an input), whilst the model structure of PK-Sim® and GastroPlus® includes the mechanistic oral absorption model to account for gut first pass impact on the systemic bioavailability of the chemicals. Other major differences in default approaches exist, such as PK-Sim® defaulting to zero renal clearance, GastroPlus® having the default be renal filtration, whilst httk defaults to renal clearance occurring at the glomerular filtration rate. The different approaches of model parameterisations can lead to varied bioavailability and explain some of the differences in *C*_max_ predictions for ouabain between the models (oral bioavailability was the default 100% in httk and was predicted to be 0.0066% in PK-Sim® and 17% in GastroPlus®). The consistently lower *C*_max_ predictions from PK-Sim® especially for 2,4,6-tri-tert-butylphenol remains unexplained in the scope of the current work.

Our PBK framework used C_max_ as the primary metric to assess systemic bioavailability, which is appropriate for acute, receptor-mediated toxicity (e.g. ouabain’s Na + /K + -ATPase inhibition, nitrobenzene’s methemoglobinemia), aligning with in vitro bioassay endpoints and in silico predictions guidance. However, for chronic toxicity (STOT-RE), AUC may be more relevant, especially for cumulative toxicity in organs such as the liver and kidneys, where prolonged exposure exacerbates “wear and tear” damage.

In our current work, we focussed on oral dosing, excluding other routes due to data limitations, modelling complexities, and scope constraints. We prioritised *C*_max_ over AUC due to limited oral STOT-RE classifications and oral route-specific data availability. Cases like chloronitrobenzene underscore the need for repeated-dose AUC analysis across various exposure routes to fully assess organ toxicity risks. This gap reflects broader challenges in bioaccumulation and its consequential impact on toxicokinetics. For comprehensive future modelling, both *C*_max_ and AUC should be treated as route-specific metrics, accounting for route-dependent ADME variations. Multi-route comparisons could also provide more complete toxicity profiles to help understand impacts of prolonged exposure on organ toxicity. To address this, the future implementation of global sensitivity analyses comparing *C*_max_/AUC-driven hepatorenal toxicity, incorporating enzyme inhibition kinetics (e.g. renal transporter competition) could be explored. Read-across models could be leveraged for chronic AUC prediction in structurally related compounds. This dual-metric approach could strengthen the current strategy by enhancing chronic risk prediction through STOT-RE endpoints.

## Overall assessment

On completion of the in silico, bioactivity, and bioavailability assessments, the bioactivity and bioavailability categories for each selected chemical were placed in the EPAA Designathon matrix, and a preliminary overall concern category is determined. This preliminary category is reviewed using a weight of evidence approach to challenge the hypothesis that all chemicals are of high concern. In essence, this aligns with the exclusion principle, and a chemical is placed into its most severe category provided there is insufficient evidence to conclude otherwise (Firman et al. 2022).

The evidence was evaluated using questions, including:

1. Has the appropriate molecule been assessed?
2. Has an indicator of toxicity been identified from in silico?
3. Does the chemical have well studied analogues?
4. Is the range of in vitro bioactivity assays sufficient (including assays appropriate for any indicator of toxicity that has been identified)?
5. Are there consistent effects at doses below cytotoxic levels (unless cytotoxicity is a lead effect at low doses)?
6. Is there consistency across the range of bioactivity assays and indicators of toxicity?
7. What is the strength of evidence supporting the bioavailability predictions: models using in silico inputs, models using in vitro inputs, or in vivo data from analogues?
8. Is there consistency across the range of bioavailability models?

These questions allow the weight of evidence to be considered when coming to a conclusion about the overall level of concern for a given chemical.

To illustrate how we integrate the three lines of evidence (bioavailability, biokinetics, and in silico), two example analyses from the original 12 chemicals, chloronitrobenzene and ouabain, will be presented.

Starting with chloronitrobenzene, the in silico assessment placed it in the High level of concern category and highlighted alerts for hepatotoxicity, haemolytic anaemia and splenotoxicity. The assessment also indicated that chloroaniline would be a major metabolite and a close mechanistic analogue aniline, suggested first by in silico and confirmed by a followed up literature research is known to also cause methemoglobinemia (Khan Firoze et al. 1993). On the basis of this information, it was decided that the bioactivity of both the parent, chloronitrobenzene, and the putative metabolite, chloroaniline had to be assessed.

Chloronitrobenzene showed no activity in 534 in vitro assays and low activity in 1 assay, the assessment deemed it to have low severity, therefore, placing it as Low in the bioactivity matrix. However, as revealed before, we may not have the appropriate range of assays as haemolytic anaemia and splenotoxicity were missing in from the in vitro assays. Furthermore, the appropriate molecule might not have been assessed, as the metabolite bioactivity still needs to be considered.

Chloroaniline, the metabolite, showed activity in a range of assays indicating effects on cytotoxicity, steroid regulation and cell proliferation (Table 2), the resulting activity matrix is shown in Table 3. These results would place chloroaniline in the Mid-level for bioactivity, but if we go back to the weight of evidence questions an appropriate assay was absent, in this case for methemoglobinemia as shown in Table 4 and in the literature (Messmer et al. 2015). Therefore, the parent, chloronitrobenzene, should be considered to have high bioactivity due to uncertainty and insufficient evidence.

The bioavailability assessment based on PBK models placed chloronitrobenzene in Mid-level (Table 6). With a high bioactivity or a mid-bioavailability level, we would place chloronitrobenzene in the overall high level of concern.

Another example of an assessment using the weight of evidence questions was ouabain. It was highlighted as having a structure related to cardiac glycoside and as such would be expected to exhibit cardiotoxicity. Ouabain showed activity in a range of assays as shown in Table 7. These results would place ouabain in the medium level of bioactivity as the one result in the high severity/high potency segment was for an oestrogen receptor assay.

**Table 7:**
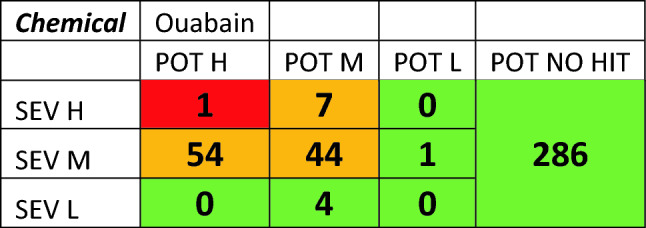
Bioactivity matrix for ouabain

Addressing the weight of evidence question concerning consistency of results (“6. Is there consistency across the range of bioactivity assays and indicators of toxicity? “) revealed that there were 5 other assays for oestrogen receptor activity that were all at the mid-level suggesting the one result in the high level was an outlier. However, the weight of evidence questions about adequate assays (“4. Is the range of in vitro bioactivity assays sufficient (including assays appropriate for any indicator of toxicity that has been identified)?”) revealed that there were no appropriate assays for cardiotoxicity in the data set. Therefore, there was insufficient evidence to move from high level for bioactivity.

The assessment for ouabain bioavailability based on PBK models placed the chemical on a low level of concern for oral exposure (Table 7). Ouabain was, therefore, considered to be a Mid overall level of concern based on high bioactivity and low bioavailability. Nevertheless, this combination highlights concerns over the type of bioavailability routes as different types of exposure (i.e. inhalation) could lead to different levels of bioavailability and physico-chemical properties and use profile would need to be considered to determine whether this would need to be addressed.

## Review of results

12 chemicals were assessed via the framework, and the initial results are shown in Table 8.

**Table 8:**
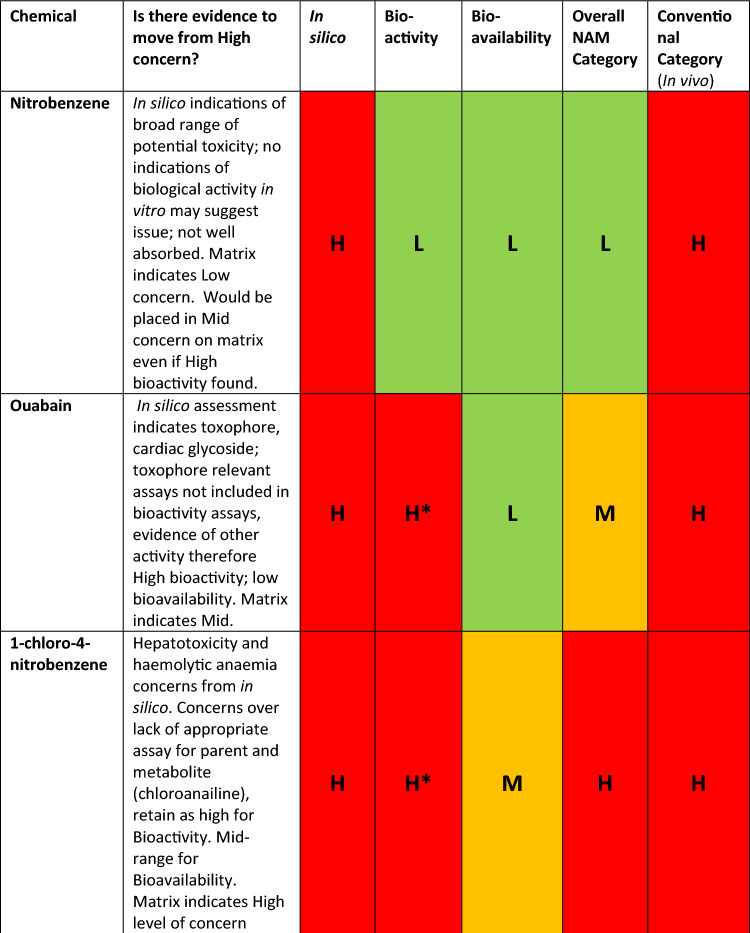

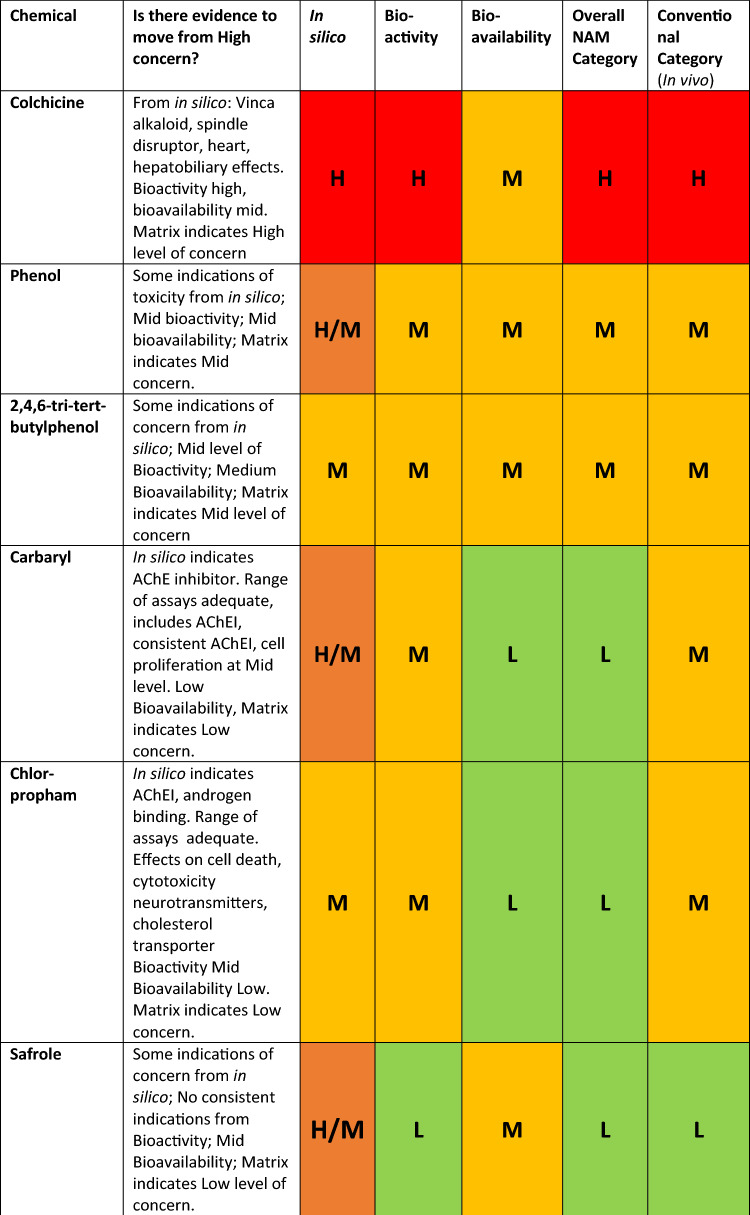

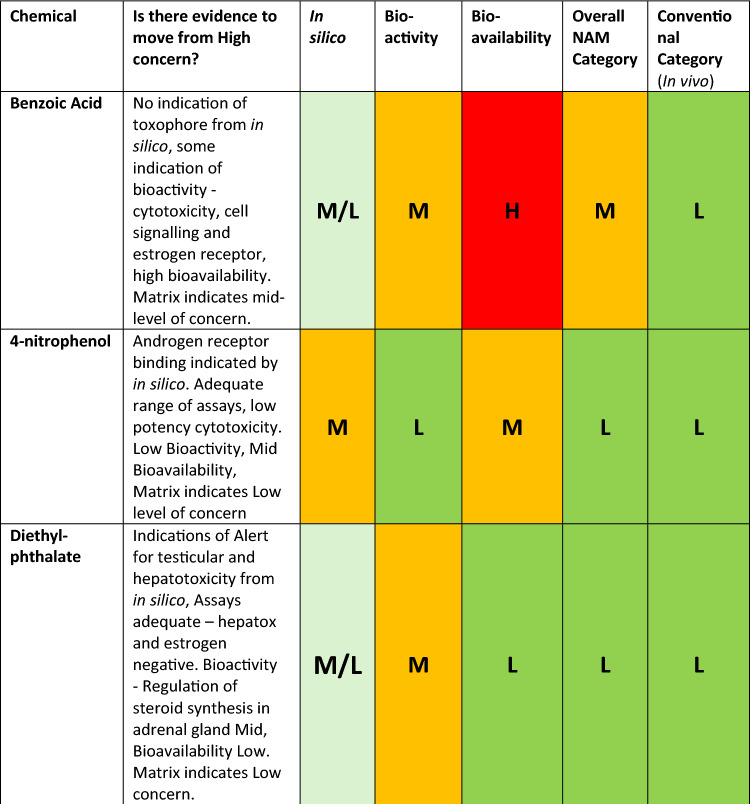
Weight of evidence and initial outcome from the assessment framework using original categorisation criteria for in vitro bioactivity and in vitro bioavailability and category derived from conventional data

H* insufficient data. Exclusionary method defaults the assessment to high

Once obtained, the ‘NAM category’ derived from the entirety of the assessment can be compared to the level of concern obtained from a conventional assessment using traditional methods, including in vivo, as seen in Table 8.

Overall, the original categorisation criteria in the framework (‘NAM category’) had a trend towards classifying chemicals in lower categories of concern compared to the ‘conventional category’ (see Table 9).

**Table 9:** Overall NAM assessment relative to the reference based on in vivo data (‘conventional category’)

Overall assessment versus conventional category		Chemical
**2 categories higher**		
**1 category higher**	**1**	**Benzoic acid**
**Same category**	**7**	**Chloronitrobenzene, colchicine, phenol, tertiary butylphenol, nitrophenol, diethylphthalate, safrole**
**1 category lower**	**3**	**Carbaryl, chlorpropham, ouabain**
**2 categories lower**	**1**	**Nitrobenzene**

The boundaries for both bioactivity and bioavailability were set using reasonable logic but they are not fixed. In the light of the trend towards lower categories of concern from NAMs assessment, it was decided to carry out a sensitivity analyses varying the criteria for categorisation of bioactivity and bioavailability, as seen in the following sections.

### Sensitivity analysis

The first sensitivity evaluation for bioactivity was to consider the category bands for potency, which were determined via AC50 values as follows: High < 0.1 µM, Mid 0.1–10 µM, Low > 10 µM. Raising these boundaries by up to a factor of 10 did not change the potency categorisation of the 12 chemicals assessed.

The second analysis investigated the effect of modifying the bioactivity matrix as shown in Table 10. The original matrix used in the previous section can be seen in Table 10a (Designated ‘Min Red’), chemicals were only assigned to High bioactivity concern when there were assays showing both high potency and high severity.

**Table 10:**
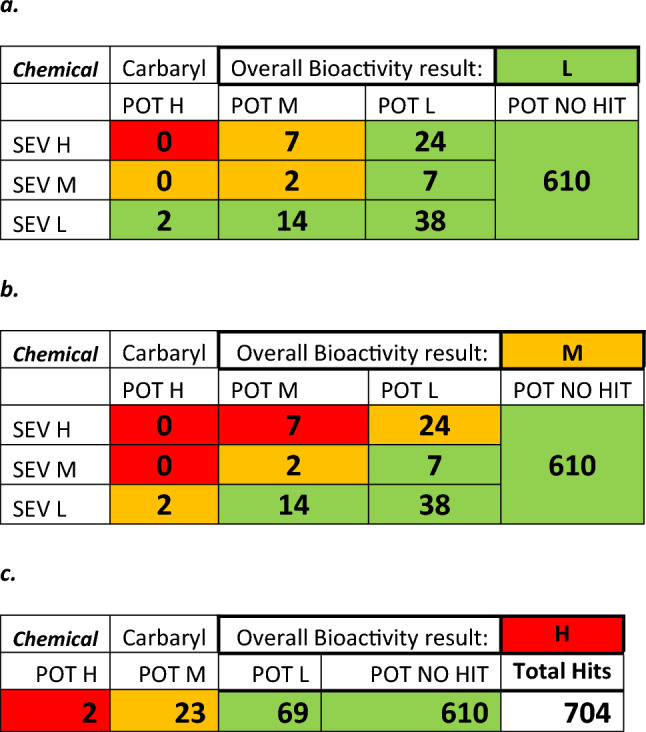
Bioactivity matrices for carbaryl: (a) The original bioactivity matrix (Min Red), (b) the revised bioactivity matrix that increased the level of concern for more cells (Max Red), and (c) the assessment based only on potency (Pot only)

The matrix was modified as shown in Table 10b, increasing the level of concern for more cells in the matrix, designated ‘Max Red’. The example shown is for carbaryl, in the original assessment it was deemed Low category for bioactivity (See Table 10a) after the sensitivity evaluation with the revised matrix it was assigned a Mid-category (Table 10b).

The next evaluation was to remove the severity assessment and categorise bioactivity only on potency (designated Pot Only). An example can be seen in Table 10c where this adjustment assigned carbaryl to the High category for bioactivity.

The next evaluation, shown in Table 11, was to assess the effect of changing the boundary criteria for bioavailability from ‘Low < 50 µM, Mid 50–500 µM, High > 500 µM’ (designated 50–500 µM) to ‘Low < 10 µM, Mid 10–100 µM, High > 100 µM’ (designated 10–100 µM). It is interesting to note that no chemicals have “low” bioavailability after this adjustment whereas with the previous limits no chemicals were regarded as having High bioavailability.

**Table 11:**
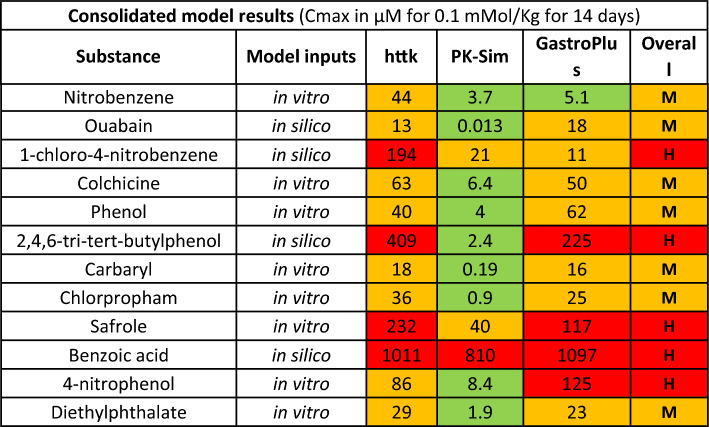
Boundary criteria change for the classification of bioavailability for the 12 selected chemicals

The overall classification is the worst-case amongst the three categories determined using three different PBK models (httk, PK-Sim, GastroPlus). High > 100 µM (red); Mid 100–10 µM (orange); Low < 10 µM (green)

**Fig. 6:**
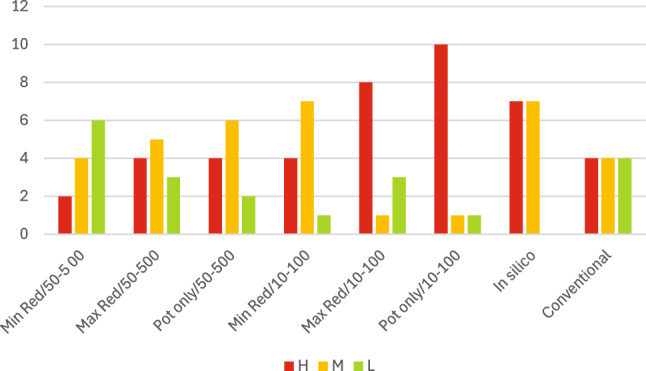
Distribution of the 12 chemicals into the three levels of concern (High, Medium, and Low) from the different adjustments of bioactivity and bioavailability. Compared to in silico and the reference level of concern (from conventional approaches using in vivo studies)

Once modifications are made to the boundary criteria, the more conservative approaches reflect a general increase in the overall level of concern. The ‘Conventional LoC’ was never considered the absolute correct answer, nor did the framework aim to get a one-to-one result. However, even if the conventional LoC results are not definitive, they are still an indication of the relative levels of concern of the 12 chemicals. Figure 6 shows the distribution of the 12 chemicals to the 3 categories derived from the different adjustments to the criteria, level of concern from the in silico analysis and the level of concern derived from the conventional approach using laboratory animal studies. The closest agreement between the conventional and NAM-based assessments was given by the “maximum red” adjustment for bioactivity coupled with the original cutoff values for bioavailability. This shows that the general approach provided by the EPAA matrix can be calibrated to the required level of protection.

## Discussion

In this section, the origins of the current classification system are reviewed and the implications for developing a NAMs based approach.

### Origins of the current classification system

The current CLP system of classification (ECHA 2023a) has 10 groupings of adverse health effects which chemicals can be placed into bands of different levels of concern. In addressing this issue, it is necessary to explore why there are 10 groupings for classification. The 10 groupings are based on a mix of adverse outcomes (AOs) and modes of action (MoA).

The AOs are divided up by.

1. Duration of exposure:
  - Acute lethality
  - STOT-SE
  - STOT-RE
  - Chronic
2. Particular organs or systems:
  - Skin and eye irritation/corrosivity
  - Sensitisation
  - Reproduction and development
3. Particular end point:
  - Carcinogenicity
4. Mode of action:
  - Mutagenicity
  - Endocrine disruption

This collection of classification divisions arose from the origins of toxicology as an observational science. Once it became clear that chemicals could cause harm to health, studies were developed to determine what potential adverse effects occur and under what conditions. The LD50 quantified how much substance was required to cause death after a single dose. In the 1940s, the use of repeated-dose studies started to answer the question of what could happen if a series of non-lethal doses were administered. Over time, the duration of these observational studies increased from 14 days, to 28 days, to 90 days, to 12 months, and to lifetime. At the same time, the breadth of observations was increased with more organs being examined histopathologically, with haematology and clinical chemistry investigations being added to give a fuller picture of the health of the laboratory animals and thus to identify more adverse outcomes.

Similarly, observational studies were developed to determine if chemicals could cause adverse effects during the life stages of reproduction and development. The observational studies based on duration of exposure were then modified to assess the end point of carcinogenesis.

As the considerable body of data from the observational studies built up, questions started to be asked about what was happening in terms of the biological mechanisms that were leading to the AOs. Gradually knowledge was gained about the changes that were taking place when chemicals come into contact with biological systems.

At the same time, the observational studies became encoded into chemical regulations aimed at protecting human health. It became an expensive and time-consuming process to develop chemicals, and there was a need to select, for development, those chemicals which would eventually prove to be acceptable. This led to the introduction of shorter term studies and in vitro assays to help the early identification of biological activity and predict the outcome of regulatory observational studies.

We have come full circle, we previously used observational studies to identify underlying biological effects, now we are using biological effects to predict the outcome of observational studies. We have a classification system based on observations from studies, which are themselves caused by biological effects, for which we have a range of assays. Could we have a classification system based on biological effects themselves without trying to predict the observations they can lead to? However, this would lead to the question of whether there is a need to differentiate between biological effects.

Hazard identification and characterisation is a means of differentiating effects and they are based on two questions:

1. What can happen?
2. How much is needed to make it happen?

The answers to these questions can be placed on a two-dimensional spectrum which can be termed “severity” and “potency”. These underline the categories within each division of the classification system. STOT encompasses both—do the observations seen pose a significant threat to health (severity), and if so, what is the No-Observed-Adverse-Effect Level, NOAEL (potency)? The classification of a substance is proportional to the determined potency value, with the most potent substances (i.e. lower NOAELs) being assigned to category 1. Category 2 is assigned to substances with a mid-range NOAEL, and no classification is assigned to substances where a NOAEL is high enough to not be a threat.

Carcinogenicity and developmental and reproductive toxicology (DART) effects are considered so severe an outcome that observations in the appropriate studies can lead to categorisation if seen below a relatively high dose set as a limit under the relevant regulations. If there is a high level of confidence that the observations are treatment related, then category 1 is used. If there is a lower level of confidence that the observations are treatment related, then category 2 is used. The categorisation process also considers the relevance of the effect observed in laboratory animals to humans by comparing modes of action.

A similar process is used for mutagenicity and for endocrine disruption.

### Incorporating severity and potency

Here arises a paradox: if we wish to reflect severity in a NAM-based classification system, we are obliged to continue to try to predict possible adverse outcomes from the biological effects we identify in the NAMs. We would then base our categories on the detection of specific biological effects at any concentration below a defined limit, selected because they indicate a sufficient level of severity. If any of these effects are identified, the chemical would be categorised as high concern (red). If these effects were not identified but other biological effects were identified, the chemical would be medium concern (orange). If no significant biological effects were identified, then the chemical would be low concern (green). Potency would not be taken into account apart from the use of limit concentrations in the studies.

On the other hand, we could have a potency-based system. We would use a range of assays to detect biological activity. We would not attempt to predict the outcome of observational studies based on the detected biological activity, and categorisation would be achieved using cutoff values based on the concentration-dependent responses seen in the assays to place the chemical into “red” for those showing activity at low doses, “orange” for those showing activity at mid-doses, and “green” for those showing little-to-no activity below a limit dose.

In the work reported in this paper, we have attempted to use both severity and potency based on information from in vitro assays to categorise chemicals.

### Defining systemic toxicity

One of the questions posed by the EPAA Designathon was whether there could be one overall classification division, which leaving aside local effects such as sensitisation and skin and eye damage, could be called “systemic toxicity”.

The aim would be to have a single category based on NAMs identifying a range of biological effects and estimating their potency. Chemicals would be placed into High, Mid, or Low levels of concern without having to correlate the biological effects identified with a particular adverse outcome. This is closer to a potency-based system than a severity-based system as outlined earlier (“Origins of the current classification”).

In the current classification system, STOT-SE and -RE are the closest to the traditional concept of “systemic toxicity”, an alternative term could be general toxicity. In fact, the STOT definition is basically anything other than carcinogenicity, mutagenicity, or reproductive toxicity (CMR). A repeat dose study of 28-day or 90-day duration is used to identify general toxicity adverse outcomes. However, there are no clear distinctions between “general toxicity” and CMR. Rather than seeing a 90-day study as a means of identifying “general toxicity”, it can be viewed as a means of identifying a wide range of biological effects. Some of the biological effects lead to changes in organs or systems that can lead to adverse outcomes. Some of these would appear in the box we label STOT, some of these biological effects could also lead to cancer in the longer term, others could lead to adverse effects on reproduction (which includes developmental toxicity within CLP). The 90-day study can identify many of the biological effects that lead to cancer, but not all—for instance mutagenicity. The 90-day study can identify many of the effects that lead to reproductive adverse outcomes, such as reproductive organ damage, but is not able to identify, for example, direct effects on the embryo.

If the same biological effects can underlie classification under different headings, then it should be expected that many chemicals would appear as category 1 under different headings. The 150 chemicals selected for study in the EPAA Designathon may be able to provide some insight into the appearance of chemicals in more than one heading. EPAA has provided a comprehensive analysis of all 150 chemical using the data provided in Annex VI of CLP (ECHA 2023a, b). 48 of these chemicals were classified as High Concern (Red) based on being in Category 1 in one or more headings in the current EU classification scheme. With most of them, 33 (69%) being listed under one heading, 11 (23%) being listed under two headings, and 4 (8%) being listed under 3 or 4 headings. The remaining 2 chemicals needed to complete the 50 were not classified by CLP and thus not considered for the following analysis.

Table 12 shows the number of chemicals in each combination of headings. It must be borne in mind that the chemicals were selected for the purpose of the Designathon and are not necessarily representative of a wider range of chemicals, which means any conclusions drawn from the data can only be tentative. In addition, the chemicals may not have been considered for all the classification headings even though there may be sufficient evidence to consider them to be of high concern. Nevertheless, the data do not support the concept of underlying biological mechanisms causing adverse outcomes across the classification headings, except perhaps for carcinogenicity. There are 13 chemicals listed under carcinogenicity, but only 3 of them appear listed only under carcinogenicity, with 8 also appearing under mutagenicity.

**Table 12:** Number of Designathon chemicals categorised as High concern in each combination of classification heading

Classification headings	Number of chemicals
**Carc**	3
**Muta**	7
**Repro**	11
STOT*	12
**Carc + Muta**	4
**Carc + Repro**	1
**Carc + STOT**	1
**Repro + STOT**	5
**Carc + Muta + Repro**	1
**Carc + Muta + Repro + STOT**	3

*Note: STOT includes acute toxicity, STOT-SE and STOT-RE as being indicative of non-CMR toxicity

### Groupings of biological mechanisms

The analysis in the previous section would lead to the suggestion that there are groups of biological mechanisms that lead to the adverse outcomes indicating high concern and that these biological mechanisms are detected and characterised by the conventional studies that are used in toxicology. Bearing in mind that the Designathon’s purpose is to explore the use of NAMs in classification, this suggests that there could be specifically designed sets of NAMs for each of the four areas of general toxicity, mutagenicity, carcinogenicity and reproductive toxicity. Table 13 shows the number of high concern chemicals that would have been identified by applying an appropriate set of assays in each of the four headings. This questions the need for a specific set of NAM assays for carcinogenicity as 94% of the chemicals would have been identified as being of high concern by sets of assays for general toxicity, reproductive toxicity and mutagenicity. There is a plausible explanation for this: mutagenicity covers genotoxic carcinogenicity and general toxicity covers non-genotoxic carcinogenicity, with reproductive toxicity covering hormonal mechanisms, a major category of non-genotoxic carcinogenicity.

**Table 13:** Number of Designathon chemicals categorised as High concern which would be identified by different combinations of test systems aimed at specific classification headings

Classification headings	Number of chemicals	Percentage of chemicals identified (out of 48)
**Carc**	13	27%
**Repro**	21	44%
**Muta**	15	31%
**STOT**	19	40%
**STOT + Muta**	32	67%
**Carc + Muta**	20	42%
**Repro + Muta**	32	67%
**Repro + STOT**	34	71%
**Carc + Repro**	29	60%
**Carc + Repro + Muta**	37	77%
**Carc + Repro + STOT**	41	85%
**Carc + STOT + Muta**	37	77%
**Repro + Muta + STOT**	45	94%

This suggests that NAM-based assessment systems should be focussed on three areas of biological effects:

- Effects leading to mutagenicity.
- Effects leading to general toxicity.
- Effects leading to reproductive (including developmental) toxicity.

The identification of significant effects at low concentrations would lead to High level of concern for bioactivity, effects at mid-concentrations would lead to Mid-level of concern, and no effects or effects at high concentrations would lead to Low levels of concern. This would require the system to be calibrated to determine how “significant”, “low”, “mid” and “high concentration” would be defined. There would be an option to retain three headings for the classification process, that would require discussion on whether downstream risk management would need to be different depending on the heading under which the chemical were to be classified.

### Can NAMs provide sufficient hazard characterisation?

The question remains as to whether non-animal NAMs are capable of providing hazard characterisation with sufficient confidence to be used in safety assessments and regulatory decision making. As so often is the case in this type of evaluation, the answer is subjective.

It depends on the strength of evidence provided by non-animal NAMs which will be different in each case. For instance, the strength of evidence will be high for chemicals with well-known structures, whereas the first chemical to be assessed in a new series will have a harder time. The process we have outlined uses the principle that all chemicals are first categorised as being of very high concern. The data are then assessed to determine whether there is sufficient evidence to classify the chemical as a medium or low concern. This could lead to some ambiguity as it appears to equate a lack of information with a high level of concern. It would be sensible to classify chemicals without sufficient evidence as “Assumed to be of high concern through lack of evidence” to make it clear.

It is also apparent that the process includes some steps which rely on expert judgement and thus could be considered subjective and make it difficult to provide the regulatory certainty that is important for both the regulator and the regulated.

It was to accommodate such issues that the ECETOC framework was developed to include the concept of staged assessment (Ball et al. 2022). The ECETOC framework is a tiered approach using TTC (Tier 0), in silico assessment (Tier 1), in vitro assessment (Tier 2) and targeted in vivo studies (Tier 3). The framework allows for decisions to be made at each tier if sufficient evidence is available for the appropriate decision being made. If there is insufficient evidence, then the assessment moves to the next tier. The EPAA Designathon aimed to examine Tier 0, Tier 1 and Tier 2 of the ECETOC framework but excluded Tier 3 which could involve targeted in vivo studies designed to take into account what had been revealed in Tiers 0–2.

It is interesting to speculate about how often it would be necessary to go to Tier 3 in assessing a chemical to provide the necessary level of confidence. It would depend on the nature of the chemical and its use case. In situations where a chemical may be required to be biologically inert, for instance in a personal care product, then it is likely that Tier 3 would not be necessary. Tier 1 and 2 (in silico/in vitro) would be capable of categorising a chemical with a sufficient level of certainty as either a High or Low levels of concern. This may be performed with enough precision to make the decision to include or exclude a chemical from a product. However, in a situation where biological activity is a must, such as a plant protection product, the likelihood of Tier 3 being required increases, especially for chemicals with novel structures. The EPAA classification scheme calls for health‐based guidance values (HBGVs) for chemicals of Mid-concern and these could be set with higher confidence following Tier 3 studies. It would also be possible for a classification in the “red” to be investigates in Tier 3 studies, although this should not be necessary in every case.

### Use of classification derived from the matrix

In this study, we have used the classification scheme suggested by the pilot phase of the EPAA Designathon to assign chemicals into three levels of concern:

- Low (L)—presumed to be non-hazardous—no further data required, can be used widely.
- Medium (M)—Hazardous chemicals—health-based guidance values (HBGVs) required, more information is needed to verify safe use.
- High (H)—chemicals of high concern—restrict use unless additional data can be provided to change the category.

These are not exact definitions and would require further consideration to determine their proper use. The current system of classification serves to provide general guidance in most instances, but specific risk management decisions are still necessary, except in cases where downstream legislation incorporates mandated risk management, such as the cutoff criteria for plant protection products.

The EPAA Designathon aims to maintain the levels of protection provided by the current system and as such it could be considered that:

- Low corresponds to not classified in the current system
- Mid corresponds to Category 2
- High corresponds to Category 1

However, it is important to note that the EPAA Designathon aim was not to reproduce the current system but to explore new approaches in classification as well as in hazard assessment methodology.

## Conclusions

In the work reported in this paper, we developed an approach based on the ECETOC framework and the EPAA matrix to differentiate between chemicals with different levels of concern based on both potency and severity. Our approach uses “cutoff” values to decide the three levels of concern (low, medium, and high) for both bioactivity and bioavailability. The use of a standardised applied dose gave rise to a single value for bioavailability that enabled us to develop a cutoff value. For this work, we limited the simulation to the oral route, but it would be possible to develop similar simulations for other exposure routes such as inhalation and dermal if the physico-chemical properties and use profile indicated that would be necessary.

We decided the cutoff values at the beginning of the process knowing that they might need to be revised as we gained more evidence from using the framework. We explored how adjusting the cutoff values changed the distribution of chemicals within the three categories. This was done after the basic properties of the chemical had been determined and the cutoff values could be further calibrated as more chemicals are assessed.

A major factor to emerge from the examples was that the process is heavily dependent on having an adequate range of assays. For example, some chemicals had an alert from the in silico assessment that could not be confirmed with the available in vitro assays. There is also uncertainty over whether the chemical or a metabolite should be assessed in vitro. Therefore, the level of confidence that the process can generate needs further thought. We used a qualitative weight of evidence approach that included the consistency of the data across multiple assays and used the in silico assessment as a means of gauging whether there was an adequate range of assays to examine the chemical. The level of confidence on this basis is higher if the chemical has a structure for which there is knowledge of its biological activity. It would be substantially more difficult to provide a proper level of confidence for a novel structure in a category of low concern.

The exclusionary principle that we used, where the chemical starts the assessment in high concern and stays there unless there is evidence to move to a category of lower concern, provides some protection against false negatives based on absence of data bit it would be important to distinguish between chemicals classified as of high concern based on sufficient evidence and those assumed to be of high concern because of insufficient evidence. Multi-constituent and UVCB (unknown or variable composition, complex reaction products or biological materials) substances will present challenges to this approach that relies to some extent on knowing the chemical structure.

Although this work on the EPAA Designathon interpreted NAMs as Non-Animal Methods, the ECETOC Framework (Ball et al 2022) considered NAMs to be new approach methodology that includes novel in vivo and ex vivo methods as part of a tiered approach.

The use of different components in a tiered approach which adds more information at each stage: e.g. in silico assessment for initial alerts, determining what biological activity the chemical may have in a range of in vitro alerting assays, and then using more specific assays to follow-up, lends itself to developing a Bayesian approach if many chemicals could be assessed to develop an algorithm. The EPAA complete list of 150 chemicals could provide the basis for such an approach.

Overall, we have demonstrated that the matrix suggested by the EPAA Designathon and ECETOC’s approach can be used to categorise chemicals into three different levels of concern but there are areas still to be explored especially for the range of assays used, the framework categorisation being defined, and how such a matrix would fit into a tiered approach, pragmatically, including targeted in vivo studies.

## Appendix A

The in silico tools and models used:

**Table:**

	Tool/model	Endpoint
Acute oral	Leadscope/stat	GHS cat (rat)
	Leadscope/expert	GHS cat (rat)
	T.E.S.T./Consensus	LD50 (rat) mg/kg
	ACD/Percepta	LD50 (rat) mg/kg
	OPERA	LD50 and classifications
	Dow acute oral toxicity model	LD50 (mg/kg) and GHS classification
Genotoxicity	Leadscope	Bacterial mut (sta)
		Bacterial mut (exp)
		In vitro Chrom Ab CHL
		In vitro Chrom Ab CHO
		In vivo Micronuc Mouse
		In vivo Chrom Ab comp
		In vivo Chrom Ab other
		In vivo Chrom Ab rat
	ACD/Percepta	Bacterial mut (sta)
		Chromosome aberrations in vitro composite
		Chromosome aberrations in vivo composite
		Micronucleus in vivo composite
	VEGA	Mutagenicity (Ames test) model (CAESAR)
		Chromosomal aberration model (CORAL)
		In vitro micronucleus activity (IRFMN-VERMEER)
		In vivo micronucleus activity (IRFMN)
	QSAR Toolbox	Bacterial OASIS alerts
		In vitro mutagenicity (Ames test) alerts by ISS
		In vivo mutagenicity (micronucleus) alerts by ISS
	TIMES Oasis	In vitro Ames
		In vitro chromosomal aberration (CA)
		In vitro mouse lymphoma (MLA)
		In vivo comet
		In vivo liver clastogenicity
		In vivo liver TGR
		In vivo micronucleus
	DEREK Nexus	Genotoxicity, mutagenicity, chromosome damage, photo-induced chromosome damage, non-specific genotoxicity and photo-induced non-specific genotoxicity
	Dow facile reactivity profiler	Facile chemical reactivity
Carcinogenicity	Leadscope	Mouse male
		Mouse female
		Rat male
		Rat female
	ACD/Percepta	Rodent
	Derek Nexus	Carcinogenicity, photocarcinogenicity,
Endocrine	Leadscope	ER bioactivation
		AR binding
		AR transactivation (antagonism)
		TPO inhibition
		TR alpha and beta transactivation
		Sodium Iodide symporter (NIS) Inhibition
	VEGA	ER-mediated effect (IRFMN-CERAPP)
		AR-mediated effect (IRFMN-CoMPARA)
	QSAR Toolbox	ER binding profiler
	OPERA	ER binding agonism/antagonism
		AR binding agonism/antagonism
Reproductive	Leadscope	Mouse male
		Mouse female
		Rat male
		Rat female
		Mouse sperm
		Rat sperm
	VEGA	Developmental/reproductive toxicity library (PG)
	DEREK Nexus	Reproductive toxicity and testicular toxicity
Developmental	T.E.S.T./Consensus	Developmental toxicant
	VEGA	Developmental toxicity (CAESAR)
		Developmental/reproductive toxicity library (PG)
	Leadscope	Foetal growth retardation
		Foetal weight decrease
		Foetal death
		Post implantation loss
		Preimplantation loss
		Structural dysmorphogenesis
		Visceral organ toxicity
	Derek Nexus	Developmental toxicity and teratogenicity
STOT	Leadscope	Heart-related effects
		Hepatobiliary effects
		Urinary tract effects
Neurotox	Leadscope	Pup behaviour
	Derek Nexus	Neurotoxicity
	Dow neuronal target models	Mechanistic neuronal targets (nAChR, mAChR, AChE, 5HT, GABA, Glycine and mitochondrial inhibition)
General TOX	QSAR Toolbox	Protein binding by OASIS
		Protein binding by OECD
		Repeated-dose HESS
	DEREK Nexus	Several related endpoints including organ toxicities, skin and respiratory sensitisation, nephrotoxicity, mitochondrial toxicity, irritation, bone marrow toxicity, bladder toxicity and other miscellaneous endpoints
	Leadscope	Human adverse cardiological effects suite
		Human adverse hepatobiliary effects suite
		Human adverse urinary effects suite
Metabolism	Meteor Nexus	Metabolism and subsequent endpoints within Derek Nexus
